# Supplementary material for: Accelerating High b-Value Diffusion-Weighted MRI Using a Convolutional Recurrent Neural Network (CRNN-DWI)
Source: Bioengineering (Basel). 2023 Jul 21;10(7):864. doi: 10.3390/bioengineering10070864 (PMC10376839; doi:10.3390/bioengineering10070864)
Supplement: Supplementary file 1 [file bioengineering-10-00864-s001.zip › bioengineering-2418660-supplementary.pdf]

# Supplementary Materials

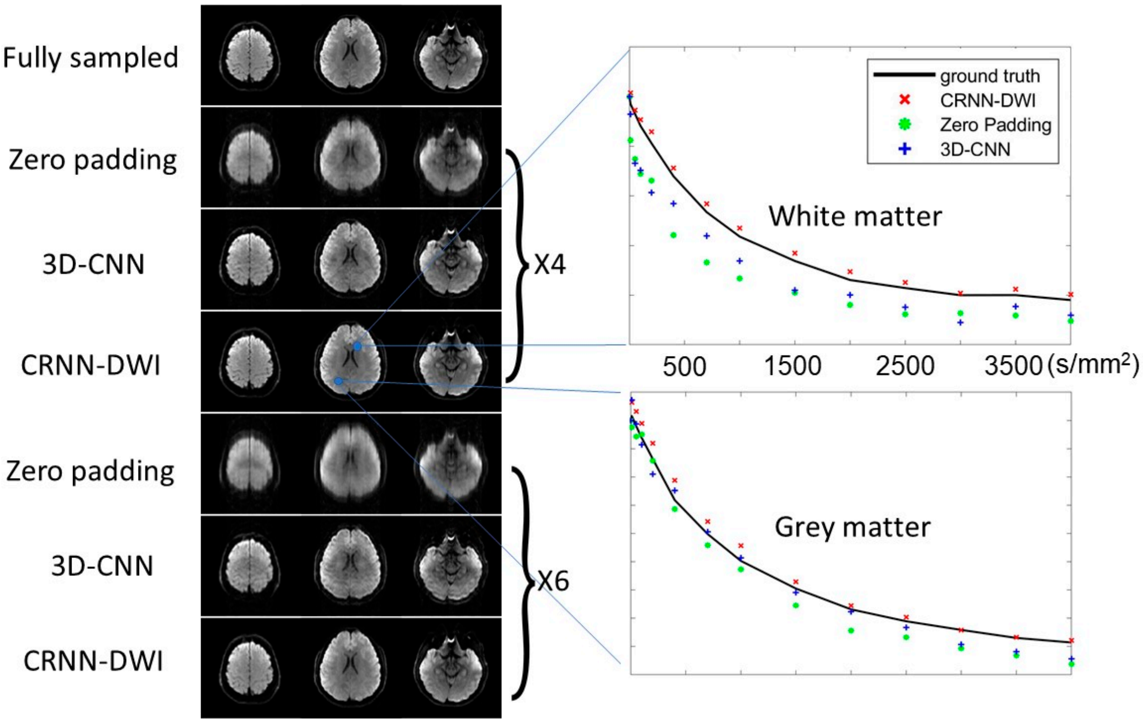


**Figure S1**. Representative trace-weighted images at b = 1000 s/mm$^2$ (left) using different reconstruction methods and the corresponding signal decay curves (right) from two randomly selected ROIs (white matter and gray matter as indicated by the blue areas). The trace-weighted images reconstructed using CRNN-DWI showed excellent image quality even with a six-fold undersampling. The signal decay curves from CRNN-DWI agreed well with the curves from the fully sampling images, whereas the curve from zero-padding and 3D-CNN exhibited substantial deviations.
